# Associations between Moderate-to-Vigorous Physical Activity and Neighbourhood Recreational Facilities: The Features of the Facilities Matter

**DOI:** 10.3390/ijerph111212594

**Published:** 2014-12-04

**Authors:** Ka Yiu Lee, Paul H. Lee, Duncan Macfarlane

**Affiliations:** 1 Institute of Human Performance, The University of Hong Kong, Pokfulam, Hong Kong, China; E-Mail: kyle2012@hku.hk; 2 School of Nursing, Hong Kong Polytechnic University, Kowloon, Hong Kong, China; E-Mail: paul.h.lee@polyu.edu.hk

**Keywords:** neighbourhood, recreational facilities, MVPA, GIS

## Abstract

*Objectives*: To examine the associations between objectively-assessed moderate-to-vigorous physical activity (MVPA) and perceived/objective measures of neighbourhood recreational facilities categorized into indoor or outdoor, public, residential or commercial facilities. The associations between facility perceptions and objectively-assessed numbers of recreational facilities were also examined. *Method*: A questionnaire was used on 480 adults to measure local facility perceptions, with 154 participants wearing ActiGraph accelerometers for ≥4 days. The objectively-assessed number of neighbourhood recreational facilities were examined using direct observations and Geographical Information System data. *Results*: Both positive and negative associations were found between MVPA and perceived/objective measures of recreational facilities. Some associations depended on whether the recreational facilities were indoor or outdoor, public or residential facilities. The objectively-assessed number of most public recreational facilities was associated with the corresponding facility perceptions, but the size of effect was generally lower than for residential recreational facilities. *Conclusions*: The objectively-assessed number of residential outdoor table tennis courts and public indoor swimming pools, the objectively-assessed presence of tennis courts and swimming pools, and the perceived presence of bike lanes and swimming pools were positive determinants of MVPA. It is suggested to categorize the recreational facilities into smaller divisions in order to identify unique associations with MVPA.

## 1. Introduction

Despite the well-reported health benefits of engaging in regular physical activity (PA) [1,2], sedentary lifestyles are still widely prevalent throughout the world [3,4]. Consequently, the promotion of a more active lifestyle has received considerable attention [5], such as in the United States where only 5% of the population met the PA recommendations [3]. Activity enhancing aspects of the built environment have the potential to influence large proportions of the population over prolonged periods [6]. These aspects of the build environment have been extensively examined [7,8,9] in an attempt to help promote PA which is known to be be effective in combating conditions such as obesity [10] and other chronic diseases [11].

The study of the how the built environment can influence PA often includes many neighbourhood characteristics such as street connectivity [12], residential density, proximity to commercial destinations, aesthetics, land use diversity [13] and in particular, the availability of recreational facilities [14] which are common destinations for leisure time physical activity [15]. A number of studies have found positive associations between PA and objectively-assessed presence of parks, walking paths and biking trails [16,17]. However, null associations [18,19], or negative associations were also found between PA and objectively-assessed presence of some types of recreational facilities [20]; see also the review by Williams *et al.* [9]. Consequently, the role of recreational facilities in promoting PA requires further investigation.

The study of recreational facilities often involves both perceived and objective measures. The perceived measures typically includes the use of self-reported questionnaires [19,21] that assess the availability of certain types of recreational facilities in the neighbourhood. The objective measures typically involve the use of systematic direct observations [22] and Geographical Information Systems (GIS) to objectively quantified the environmental variables [7]. Making use of these measures, it has been common to categorize neighbourhood recreational facilities into several different types, such as the basketball courts, bike trails and walking paths [18,19], and their presence and number are then quantified in each area. However, the literature informs us that the mere presence and number of recreational facilities may not be sufficient to support PA, since other features of these recreational facilities (e.g., low costs and good lighting) may also determine facility use [21] and PA [20]. Furthermore, outdoor and indoor recreational facilities may have different impacts on facility use and PA. For example, outdoor activities are restricted by the weather and safety issues [19], making indoor activities more preferable under some circumstances (e.g., bad weather and night time). In contrast, indoor activities are immune from the influence of the external environment although there may be less favourable external aesthetic features (e.g., natural landscapes).

Differences may also exist between public, residential and commercial recreational facilities in terms of their proximity to residences and costs of use. For example, residential recreational facilities in Hong Kong are typically easily accessible as they are located within private residential complexes (typically multiple apartment tower-blocks), unlike the more remote access provided by public and commercial recreational facilities. However, public recreational facilities may provide the lowest facility user costs than residential and commercial recreational facilities, at the later often both require users to pay for memberships. The overall perceived quality of public, residential and commercial recreational facilities may also differ considerably, depending on the nature of their management policies. Consequently, categorizing each type of recreational facility into different usage locations would be essential in order to identify their unique associations with PA. Although Ries *et al*. [23] investigated the role of public *versus* private recreational facilities on PA in urban youth, they did not look into the role played by each specific type of facility. Whilst Eriksson *et al*. [24] were able to obtain geocoded data showing nine different categories of exercise facilities, their analysis only compared the total number of facilities available (≥4, 1–3, 0) against accrued moderate-to-vigorous PA (MVPA). Thus, to the best of our knowledge, no previous studies have examined the effect of categorizing a wider range of 14 different types of common recreational facilities (especially those public facilities managed by Hong Kong’s Leisure and Cultural Services Department and often found in many neighborhoods), into six different combinations of usage location: indoor *versus* outdoor venues, and across public *versus* residential *versus* commercial recreational facilities, and then comparing their individual effect on MVPA.

Some evidence has shown that the perceived presence of recreational facilities, but not the objectively-assessed presence nor their number, have been determinants of PA [18,19], implying that facilities perceived by residents may have a more important role in determining PA than the objective physical environment [25]. Despite the importance of facility perceptions, few studies have examined how the facility perceptions match with the corresponding objectively-assessed number of public, residential and commercial recreational facilities available in the neighbourhood. Scott *et al*. [19] examined the associations between facility perceptions and objectively-assessed number of recreational facilities at two levels of proximity, but the examined facilities only included public and commercial recreational facilities. Consequently, it remains uncertain how Hong Kong’s somewhat unique availability of public, residential and commercial recreational facilities across a range of neighbourhoods are associated with perceived facilities by the residents.

Filling in these clear research gaps, this study aimed to examine the associations between objectively-assessed moderate-to-vigorous physical activity (MVPA) and both the perceived and the objective availability of neighbourhood recreational facilities categorized into six different combinations of usage locations: public indoor/outdoor; residential indoor/outdoor; commercial indoor/outdoor. The study also aimed to examine the associations between individual perceived facilities (e.g., perceived presence of basketball courts) and their corresponding objective number of public, residential and commercial recreational facilities (e.g., objectively-assessed number of public basketball courts).

It was hypothesized that objective and perceived measures of some types of recreational facilities, including basketball courts, walking paths and bike trails, would be positively associated with MVPA, but their features (indoor *versus* outdoor, public *versus* residential or commercial) would determine the significance of associations. Having a higher objectively-assessed number of recreational facilities available in the neighbourhood was hypothesized to increase the odds of perceived availability of the corresponding facilities, and with greater odds of perceived facility availability expected for existing residential recreational facilities due to their greater proximity to residences.

## 2. Experimental Section

### 2.1. Sampling Areas

This cross-sectional study was conducted within January 2008–December 2011 in Hong Kong, an ultra-dense Asian city where the territory is divided into 197 small divisions termed ‘tertiary planning units’ (TPUs). A total of 28 TPUs were selected in this study and they represent four types of neighbourhoods: (a) high walkability and low socioeconomic status, (b) high walkability and high socioeconomic status, (c) low walkability and low socioeconomic status and (d) low walkability and high socioeconomic status. A total of eight blocks of TPUs were selected in each of the four types of neighbourhoods, leading to a total of 32 neighbourhoods being selected. In each selected neighbourhood, one residential building was randomly sampled to recruit participants.

The walkability indices of the TPUs were determined by household density and street intersection density obtained from the Geographical Information System (GIS). The calculations of the indices were accomplished based on the formula [26]:

Walkability index = (HD – HDM)/HDSD + (ND – NDM)/NDSD (1)

where HD = household density; HDM = mean of household density; HDSD = standard deviation of household density; ND = street intersection density; NDM = mean of street intersection density; NDSD = standard deviation of street intersection density. The TPUs having the walkability indices above and below zero were considered having ‘high’ and ‘low’ walkability respectively.

Data regarding the neighbourhood socioeconomic status (SES) were obtained from the Census and Statistics Department. Households with a monthly income greater than HKD $25,000 was considered a ‘high income household’, whilst a household with a monthly income lower than HKD $10,000 was considered a ‘low income household’ [27]. A neighbourhood was considered as ‘high SES’ when the high income household ratio was greater than 50% and the low income household ratio was less than 15%. In contrast, a neighbourhood was considered as ‘low SES’ when the high income household ratio was less than 30% and the low income household ratio was greater than 25%. These selection criteria of neighbourhoods were based on the Hong Kong Census data in 2006 and were adopted in another study that developed a Hong Kong environmental audit tool [28].

### 2.2. Participants

This study aimed to recruit 480 participants aged 18–65 years to complete an interviewer-administered questionnaire through face-to-face interviews and a sub-sample of 200 was randomly chosen and asked to wear an accelerometer for seven consecutive days. Recruitment letters were posted to the selected residential buildings, with the aims of this study, requirements for participation and experimenter contact information provided. Residents who demonstrated their interest in participation and met the study criteria were informed the date and time for conducting the interviews. A total of 480 participants completed the questionnaires and 190 of them wore an accelerometer (10 refused; of the remainder, 154 provided valid accelerometry data—see Section 2.6). Written informed consent was obtained from all participants before participation and ethical approval for this study was obtained from the University of Hong Kong and performed in accordance with the Declaration of Helsinki.

### 2.3. Objective Measures of PA

ActiGraph uniaxial accelerometers (Actitrainer model; size of 8.6 × 3.3 × 1.5 cm; weight 51.0 g) were used to measure the PA of the participants. Following the standard practice in accelerometer-based studies for adults [29,30], an epoch length of 60 s was selected in the initialization process. The accelerometers were then threaded onto a belt which and located at the mid-axillary line at the right hip. Participants were given face-to-face instructions for proper attachment and were requested to wear the accelerometers during waking hours except during aquatic activities for seven consecutive days. No follow-up contact was made over this period and after seven days the collection of each accelerometer was performed by the experimenter at a location chosen by the participant.

PA was quantified and expressed in terms of numerical ‘activity counts’ which were categorized into different intensities of PA based on Metzger *et al.* cut points [29]. Activity counts greater than 2020 counts per minute were considered MVPA and the outcome variable was expressed as the average minutes per day spent on MVPA.

### 2.4. Objective Measures of Recreational Facilities inside the Neighbourhoods

Direct observations and data obtained from the Geographical Information System (GIS) were used to objectively measure the number of recreational facilities in the examined neighbourhoods, which were restricted within a 400-metre radial buffer area [12,18,31] originated at the centre of each selected residential buildings (geocoded from the coordinates given by www.CentaMap.com, 2007). The GIS database used was under the ‘facility layer’ of B5000 (1:5000) base map of the Hong Kong Lands Department (version 2005), using ESRI ArcGIS 8.0 and Arc View 3.1 at the GIS laboratory at the Geography Department, Hong Kong University. A trained auditor was assigned to the examined neighbourhoods to conduct direct observations and record the number of different types of recreational facilities with different features, including the indoor and outdoor, public, residential and commercial recreational facilities. Public recreational facilities refer to those open to public access and are mostly managed by a government department, the Leisure and Cultural Services Department, with a low (subsidized) usage fee. In contrast, residential recreational facilities refer to those private facilities accessible to those living in these large multistory residential complexes and often have a residents-only clubhouse containing a variety of recreational facilities. Commercial recreational facilities are private facilities that are accessible only to those who have paid a private membership, for example California Fitness or Pure Fitness. During the direct observations, the types of recreational facilities observed in the examined neighbourhoods included tennis courts, table tennis courts, badminton courts, basketball courts, volleyball courts, soccer pitches, squash courts, walking trails, bike lanes, playgrounds for children, weight and cardio training gyms, sport climbing walls and swimming pools, with their features and number recorded (Table 1). In contrast, data obtained from the GIS only included the number of parks, pavilions, sport centres, bike lanes, multi-purpose playgrounds, swimming pools and sport grounds. The GIS database therefore only reported the total number of each facility in the 32 neighborhoods in Table 1, but it was not capable of determining if the facility was indoor *versus* outdoor, nor if it was residential, public, or commercial, hence “N/A” is recorded in Table 1. To determine the presence or absence of each type of recreational facility in the analysis, the objectively-determined number of individual types of recreational facilities were dichotomized as being 1 = objective presence (if one or more facility) or 0 = objective absence (if no facility).

**Table 1:** Total number of recreational facilities across different types and features across all the 32 selected neighbourhoods measured by direct observations and Geographical Information System (GIS).

Type of Facilities	PO *	RO	CO	PI	RI	CI	Total
**Direct Observations**							
Playgrounds for Children	87	17	0	5	1	2	112
Tennis Courts	44	11	33	0	0	1	89
Badminton Courts	5	11	0	62	1	3	82
Basketball Courts	47	4	1	16	0	1	69
Table Tennis Courts	2	2	0	34	1	13	52
Squash Courts	0	0	0	39	5	8	52
Soccer Pitches	35	0	1	0	0	0	36
Volleyball Courts	13	0	0	13	0	1	27
Walking Trails	23	0	0	0	0	0	23
Weight Training Gyms	0	0	0	7	3	11	21
Cardio Training Gyms	0	0	0	7	3	11	21
Swimming Pools	5	6	3	2	0	1	17
Bike Lanes	3	0	0	0	0	0	3
Sports Climbing Walls	0	0	0	2	0	0	2
**GIS-Based**							
Sports Grounds	NA	NA	NA	NA	NA	NA	67
Pavilions	NA	NA	NA	NA	NA	NA	64
Parks	NA	NA	NA	NA	NA	NA	58
Multi-purpose Playgrounds	NA	NA	NA	NA	NA	NA	56
Swimming Pools	NA	NA	NA	NA	NA	NA	16
Sports Centres	NA	NA	NA	NA	NA	NA	11
Bike Lanes	NA	NA	NA	NA	NA	NA	1

* PO = Public Outdoor; RO = Residential Outdoor; CO = Commercial Outdoor; PI = Public Indoor; RI = Residential Indoor; CI = Commercial Indoor; NA = Not applicable as no separate categorization of features (PO, PI, *etc*) was provided in the GIS database, only the total number of facilities.

### 2.5. Perceived Presence of Recreational Facilities inside the Neighbourhoods

The perceived presence of recreational facilities was measured using a simple dichotomous scale (Yes/No questions) in which participants were asked whether the following 14 types of recreational facilities, regardless of their number and usage location, were available within 15-minute walking distance from their residence [32]: tennis courts, table tennis courts, badminton courts, basketball courts, volleyball courts, soccer pitches, squash courts, walking trails, bike lanes, playgrounds for children, weight and cardio training gyms, sport climbing walls and swimming pools.

### 2.6. Accelerometer Data Cleaning and Processing

The accelerometer data were downloaded using the ActiGraph software version 3.2.2. As many participants did not provide the requested seven full days of data, at least 10 h per day of accelerometer-wearing over at least three weekdays plus one weekend was considered sufficiently valid data for analysis [3]. The total accelerometer-wearing hours were calculated by subtracting the non-wearing hours from the total recorded hours that were the intervals between the first non-zero activity counts and the last. The periods of consecutive 30 min or above showing ‘zero activity counts’ [33,34] were considered non-wearing time periods. A total of 154 out of 190 participants (81.1%; 10 refusals) provided valid accelerometer data for the analyses. The resulting accelerometer data were positively skewed and thus square root transformations were applied to maximize normality. The subsequent statistical analyses were based on the square root of the average minutes per day spent on MVPA as the outcome variable.

### 2.7. Statistical Analyses

For the initial three analysis models (first, second, final), generalized estimating equations with robust standard error adjusting for the neighbourhood clustering effect were used to analyze the associations between the perceived/objective measures of recreational facilities and the transformed MVPA. The first model estimated the relationships of each single environmental predictor with the transformed MVPA (the environmental predictor was the presence and number of each recreational facility). All significant (*p* < 0.05) environmental predictors from the first model were included in a second multiple-predictor model. All environmental predictors that remained significant from the second model were included in the final multiple-predictor model. The associations between (a) MVPA and the objectively-assessed number of recreational facilities (Section 3.1); and (b) MVPA and both the perceived and the objectively-assessed presence of a facility (Section 3.2), were each analyzed in separate models. Data obtained from direct observations (audit) and by the Geographical Information System were also analyzed separately.

Binary logistic regression adjusting for the neighbourhood clustering effect was used to predict individual facility perceptions (perceived presence and absence) using the corresponding objectively-assessed number of public, residential and commercial recreational facilities available in the neighbourhood (Section 3.3). Confounders, including the socio-demographics of participants, the neighbourhood walkability and socioeconomic status were adjusted in the above analyses. SPSS version 18.0 was used to complete the above data analyses.

## 3. Results

A total of 64 males and 90 females (*n* = 154) with the mean age of 42.7 years (SD = 12.7) provided both questionnaires and valid accelerometer data. More than half of the participants were tertiary educated and almost 54% of them had a household income of at least HKD $25,000 per month. The average time spent on MVPA per day was 44.4 min (SD = 25.7). The socio-demographic characteristics of participants who provided both valid accelerometer and questionnaire data are presented in Table 2.

**Table 2:** Socio-demographic characteristics of 154 participants who provided both valid questionnaire and accelerometer data.

Characteristics	Neighbourhood Types #				
	HW/LSES	HW/HSES	LW/LSES	LW/HSES	Total
Male, Number (%)	24 (15.6)	11 (7.1)	7 (4.5)	22 (14.3)	64 (41.6)
Female, Number (%)	23 (14.9)	25 (16.2)	12 (7.8)	30 (19.5)	90 (58.4)
Age, Mean (SD)	46.1 (11.6)	40.4 (13.6)	44.7 (12.5)	40.5 (12.7)	42.7 (12.7)
Education, Number (%) *					
Primary or below	3 (2.0)	1 (0.7)	5 (3.3)	1 (0.7)	10 (6.5)
Secondary	26 (17.0)	8 (5.2)	6 (3.9)	16 (10.5)	56 (36.6)
Tertiary or above	18 (11.8)	26 (17.0)	8 (5.2)	35 (22.9)	87 (56.9)
Marital Status, Number (%) *					
Single and living by him/herself	2 (1.3)	3 (2.0)	4 (2.6)	4 (2.6)	13 (8.5)
Single and living with friends or relatives or family	11 (7.2)	13 (8.5)	6 (3.9)	14 (9.2)	44 (28.8)
Single parent living with one or more children	5 (3.3)	2 (1.3)	0 (0)	1 (0.7)	8 (5.2)
Couple (married or cohabit) living with one or more children	26 (17.0)	12 (7.8)	8 (5.2)	20 (13.1)	66 (43.1)
Couple (married or cohabit) living with no children	3 (2.0)	5 (3.3)	1 (0.7)	13 (8.5)	22 (14.4)
**Employment, Number (%)**					
Employed	36 (23.4)	19 (12.3)	13 (8.4)	31 (20.1)	99 (64.3)
Unemployed	11 (7.1)	17 (11.0)	6 (3.9)	21 (13.6)	55 (35.7)
Monthly Household Income in Hong Kong dollars, Number (%)*					
<$10,000	10 (6.5)	7 (4.5)	8 (5.2)	4 (2.6)	29 (18.8)
$10000–24,999	18 (11.7)	7 (4.5)	6 (3.9)	10 (6.5)	41 (26.6)
>$24,999	19 (12.3)	21 (13.6)	5 (3.2)	38 (24.7)	83 (53.9)

# H = High; L = Low; W = Walkability; SES = Socioeconomic status; * Missing values for Education (N = 1), Marital Status (N = 1) and Monthly Household Income (N = 1).

### 3.1. Associations between MVPA and Objectively-Assessed Number of Recreational Facilities

The first single-predictor model shows that both positive and negative associations were found between MVPA and the single environmental predictors regarding the objectively-assessed number of recreational facilities across different types and features, as measured by direct observations. Adjusting for the socio-demographics and neighbourhood types, the objectively-assessed number of residential outdoor table tennis courts, public outdoor badminton courts and walking trails, public indoor playgrounds for children and public indoor swimming pools were positively associated with MVPA (Table 3). In contrast, the residential indoor table tennis courts, badminton courts, squash courts, playgrounds for children, weight and cardio training gyms, public outdoor basketball courts and GIS-based multi-purpose playgrounds were negatively associated with MVPA.

**Table 3:** Associations between objectively-assessed moderate-to-vigorous physical activity (MVPA) and the objectively-assessed number of neighbourhood recreational facilities across different types and features measured by direct observations and Geographical Information System (GIS).

Predictors of MVPA	First Model with Single Predictor	Second Model with Multiple Predictors	Final Model with Multiple Predictors
	B Coefficient ^ (95% CI)	B Coefficient ^ (95% CI)	B Coefficient ^ (95% CI)
**Direct Observations**			
**Table Tennis Courts**			
Residential Outdoor	3.00 (1.91, 4.095) ***	2.44 (0.986, 3.884) **	2.80 (1.674, 3.922) ***
Residential Indoor	−1.03 (−1.486, −0.578) ***	−0.06 (−0.665, 0.536)	NA
**Badminton Courts**			
Public Outdoor	0.38 (0.001, 0.751) *	0.65 (−0.503, 1.793)	NA
Residential Indoor	−1.03 (−1.486, −0.578) ***	−0.06 (−0.665, 0.536)	NA
**Basketball Courts**			
Public Outdoor	−0.22 (−0.371, −0.061) **	−0.04 (−0.304, 0.232)	NA
**Squash Courts**			
Residential Indoor	−1.04 (−1.323, −0.75) ***	−0.68 (−1.362, 0.008)	NA
**Walking Trails**			
Public Outdoor	0.26 (0.007, 0.515) *	0.30 (−0.001, 0.608)	NA
**Playgrounds For Children**			
Public Indoor	0.62 (0.015, 1.224) *	−0.76 (−1.940, 0.420)	NA
Residential Indoor	−1.03 (−1.486, −0.578) ***	−0.06 (−0.665, 0.536)	NA
**Weight Training Gyms**			
Residential Indoor	−1.31 (−1.695, −0.92) ***	−0.30 (−1.561, 0.971)	NA
**Cardio Training Gyms**			
Residential Indoor	−1.31 (−1.695, −0.92) ***	−0.30 (−1.561, 0.971)	NA
**Swimming Pools**			
Public Indoor	0.86 (0.2, 1.515) *	1.75 (0.187, 3.319) *	0.80 (0.138, 1.469) *
**GIS-Based**			
**Multi-purpose Playgrounds**	−0.24 (−0.425, −0.062) **	−0.24 (−0.425, −0.062) **	−0.24 (−0.425, −0.062) **

^ Adjusted for age, gender, education, monthly household income, employment, marital status, neighbourhood walkability and socio-economic status; * *p* < 0.05; ** *p* < 0.01; *** *p* < 0.001; NA = Not applicable as non-significant results found in the second multiple-predictor models.

The residential outdoor table tennis courts (b = 2.80; 95% CI: 1.674, 3.922), public indoor swimming pools (b = 0.80; 95% CI: 0.138, 1.469) and GIS-based multi-purpose playgrounds (b = −0.24; 95% CI: −0.425, −0.062) remained significant in the final multiple-predictor model (Table 3). No other types or features of commercial recreational facilities, nor the GIS data including the objectively-assessed number of parks, pavilions, sport centres, bike lanes, swimming pools and sport grounds were associated with MVPA in the final multiple-predictor model.

### 3.2. Associations between MVPA and Perceived/Objectively-Assessed Presence of Recreational Facilities

The first single-predictor model (Table 4) shows that both positive and negative associations were found between MVPA and the single environmental predictors regarding the perceived or objectively-assessed presence of recreational facilities. The perceived presence of bike lanes and swimming pools, the objectively-assessed presence of tennis courts, swimming pools, and the GIS-based parks and pavilions were positively associated with MVPA, whilst the objectively-assessed presence of soccer pitches and squash courts, and the GIS-based multi-purpose playgrounds were negatively associated with MVPA in the first single-predictor model. All the above predictors remained significant in the final multi-predictor model except for the objectively-assessed presence of GIS-based parks (Table 4).

**Table 4:** Associations between objectively-assessed moderate-to-vigorous physical activity (MVPA) and perceived/ objectively-assessed presence of recreational facilities measured by questionnaire, direct observations and Geographical Information System (GIS).

Predictors of MVPA	First Model with Single Predictor	Second Model with Multiple Predictors	Final Model with Multiple Predictors
	B Coefficient ^ (95% CI)	B Coefficient ^ (95% CI)	B Coefficient ^ (95% CI)
**Perceived presence of recreational facilities (Measured by questionnaire)**			
Bike Lanes	0.80 (0.205, 1.394) **	0.73 (0.176, 1.288) *	0.73 (0.176, 1.288) *
Swimming Pools	0.78 (0.194, 1.368) **	0.62 (0.116, 1.120) *	0.62 (0.116, 1.120) *
**Objectively-assessed presence of recreational facilities (Measured by direct observations)**			
Tennis Courts	0.63 (0.075, 1.19) *	0.64 (0.155, 1.126) *	0.64 (0.155, 1.126) *
Soccer Pitches	−1.07 (−1.591, −0.546) ***	−0.88 (−1.331, −0.419) ***	−0.88 (−1.331, −0.419) ***
Squash Courts	−0.59 (−0.949, −0.224) **	−0.41 (−0.688, −0.121) **	−0.41 (−0.688, −0.121) **
Swimming Pools	0.72 (0.074, 1.357) *	0.60 (0.022, 1.181) *	0.60 (0.022, 1.181) *
**Objectively-assessed presence of recreational facilities (Measured by GIS)**			
Parks	0.68 (0.007, 1.342) *	0.22 (−0.427, 0.862)	NA
Pavilions	0.89 (0.053, 1.721) *	0.85 (0.012, 1.687) *	0.93 (0.071, 1.783) *
Multi-purpose Playgrounds	−0.72 (−1.396, −0.052) *	−0.69 (−1.361, −0.016) *	−0.75 (−1.348, −0.145) *

^ Adjusted for age, gender, education, monthly household income, employment, marital status, neighbourhood walkability and socio-economic status; * *p* < 0.05; ** *p* < 0.01; *** *p* < 0.001; NA = Not applicable as non-significant results found in the second multiple-predictor models.

### 3.3. Associations between Perceived Presence of Recreational Facilities and the Corresponding Objectively-Assessed Number

Table 5 shows that the perceived presence of all 14 types of recreational facilities was positively associated with the corresponding total objectively-assessed number of recreational facilities measured by direct observations. The objectively-assessed number of all types of public recreational facilities was associated with the corresponding facility perceptions except for the cardio training gyms. Some associations were dependent on whether the recreational facilities were public, residential or commercial facilities. The objectively-assessed number of public basketball courts, squash courts and swimming pools were positively associated with the corresponding facility perceptions but the associations were not found in residential and commercial facilities. Similarly, the objectively-assessed number of commercial cardio training gyms was positively associated with the corresponding facility perceptions but the associations were not found in public and residential facilities.

**Table 5:** Associations between perceived presence of recreational facilities and the corresponding objectively-assessed number of recreational facilities measured by direct observations.

Odds Ratio (95% CI) ^				
Perceived Recreational Facilities	Objective Quantities of Public Facilities	Objective Quantities of Residential Facilities	Objective Quantities of Commercial Facilities	Objective Quantities of Total Facilities
Tennis Courts	1.45 (1.235, 1.689) ***	NS	1.16 (1.013, 1.320) *	1.34 (1.167, 1.548) ***
Table Tennis Courts	1.31 (1.106, 1.562) **	16.15 (6.624, 39.37) ***	NS	1.32 (1.134, 1.543) ***
Badminton Courts	1.20 (1.099, 1.302) ***	1.80 (1.331, 2.427) ***	NS	1.29 (1.183, 1.396) ***
Basketball Courts	1.66 (1.291, 2.131) ***	NS	NS	1.51 (1.223, 1.868) ***
Volleyball Courts	1.67 (1.283, 2.159) ***	N/A	NS	1.74 (1.333, 2.266) ***
Soccer Pitches	1.31 (1.037, 1.644) *	N/A	NS	1.28 (1.029, 1.591) *
Squash Courts	1.20 (1.080, 1.324) **	NS	NS	1.19 (1.082, 1.309) ***
Walking Trails	1.40 (1.068, 1.843) *	N/A	N/A	1.40 (1.068, 1.843) *
Bike Lanes	69.76(19.397, 250.905) ***	N/A	N/A	69.76(19.397,250.905) ***
Playgrounds for Children	1.63 (1.184, 2.233) **	1.85 (1.041, 3.274) *	N/A	2.04 (1.498, 2.789) ***
Weight Training Gyms	1.97 (1.101, 3.534) *	NS	1.42 (1.021, 1.987) *	1.63 (1.189, 2.23) **
Cardio Training Gyms	NS	NS	1.70 (1.185, 2.425) **	1.96 (1.397, 2.747) ***
Sport Climbing Walls	3.26 (1.329, 7.989) *	N/A	N/A	3.26 (1.329, 7.989) *
Swimming Pools	11.04 (4.443, 27.422) ***	NS	NS	5.36 (2.786, 10.321) ***

^ Adjusted for age, gender, education, monthly household income, employment, marital status, neighbourhood walkability and socio-economic status; * *p* < 0.05; ** *p* < 0.01; *** *p* < 0.001; NS = Non-significant; N/A = Not applicable due to no variations across neighbourhoods.

## 4. Discussion

The findings of this study supports the hypothesis that both the perceived and objective measures of recreational facilities are important determinants of MVPA and some associations were dependent on whether the recreational facilities were indoor or outdoor, public or residential facilities. Non-significant associations have been found between PA and objectively-assessed number of recreational facilities in some previous studies [18,19] which commonly categorized recreational facilities into mere types (e.g., basketball courts) without accounting for their environment (indoor *versus* outdoor), their usage costs, or their proximity to residences (residential *versus* public or commercial facilities). Although no cause and effect relationships could be inferred in this cross-sectional study, the unique associations found between MVPA and recreational facilities across different types and features indicates that categorizing recreational facilities into smaller divisions based on their usage locations are necessary in order to identify their potential role in determining levels of MVPA.

The findings support that the objectively-assessed number of residential outdoor table tennis courts and public indoor swimming pools, and the objectively-assessed presence of tennis courts, swimming pools and pavilions were important determinants of MVPA, implying that these types and features of recreational facilities available inside the neighbourhoods may increase the likelihood of neighbourhood residents increasing their levels of MVPA. Other researchers such as Hino *et al*. [35] and Ericksson *et al*. [24] have also found that a higher number of objectively-determined total exercise facilities can promote MVPA, however, to our knowledge no studies have looked at which specific recreational facilities appear to have the greatest ability to promote PA.

It is worth noting that residential recreational facilities are typically located closer to residences than are public recreational facilities, but the former often require memberships and hence higher costs. However, we observed that most residential recreational facilities were available in high socio-economic status neighbourhoods where people were more likely to afford these costs. Consequently, although residential facilities typically require higher costs, the presence of closer (yet more expensive) residential facilities such as table tennis courts may gain an advantage over distant public table tennis courts in determining MVPA, as the former are more accessible. Sallis *et al*. [36] also found that people having more expensive recreational facilities near their home reported more engagement in exercise, suggesting having close proximity to recreational facilities, regardless of the costs, is important in promoting PA. Given that public, residential and commercial recreational facilities have different degrees of proximity to residents and also different costs, further investigations would be needed to compare the interactions of cost and proximity on how public, residential and commercial recreational facilities influence MVPA.

It is of interest to observe that public indoor, but not outdoor swimming pools were determinants of MVPA. In Hong Kong, public outdoor and indoor swimming pools require the same costs (fees set by the Leisure and Cultural Services Department, HKSAR). The associations found between MVPA and public indoor swimming pools may be indicative that indoor environments are preferred for particular sport activities due to personal preferences, weather and safety issues [19]. Contributing to the likely benefits of indoor facilities is the fact that in Kong Kong there are strong negative attitudes and behavior patterns against the high levels of ambient sunlight [37]. However, the findings do not necessarily infer that participants accrued more MVPA by using these swimming-based recreational facilities, especially since the participants were told to remove the accelerometers during aquatic activities. It is also believed that the presence of any recreational facilities may enhance MVPA simply by increasing the opportunities for transportation activities in the neighbourhood [20], especially the presence of pavilions in Hong Kong, which provide a resting place for longer walking journeys. The presence of recreational facilities may also improve the overall neighbourhood aesthetics and social cohesion, which in turn may encourage MVPA in the neighbourhood.

The negative associations found between MVPA and objectively-assessed presence of soccer pitches, squash courts and GIS-based multi-purpose playgrounds, and the objectively-assessed number of GIS-based multi-purpose playgrounds indicated that these types of recreational facility may discourage MVPA. Cohen *et al.* [20] also found negative associations with the skateboard areas, lawn games courts and golf courses, implying that certain types of recreational facilities may be unfavourable to PA. For some activities this is understandable, such as skateboarding, which is often considered as an anti-social behaviour [38]. However, it is unclear that how the presence of soccer pitches, squash courts and multi-purpose playgrounds hinder MVPA and thus further investigations of other factors would be needed, including the social environment and crime safety near and within these recreational facilities where males in particular may dominate these sports/areas.

Consistent with the findings of Hoehner *et al.* [18] the perceived presence of bike lanes was positively associated with MVPA. However, neither the objectively-assessed number nor presence of bike lanes showed significant associations with MVPA. The findings implied that perceived presence of facilities such as bike lanes were more important in determining MVPA than were the objectively-assessed physical environment, as neighborhood residents are clearly unlikely to use an existing facility for MVPA if they are unaware of its presence. As a consequence, policy makers should enhance the public awareness of recreational facilities in the built environments where they exist in an attempt to promote additional MVPA accrued by the residents.

The findings also support the contention that having higher numbers of objectively-assessed recreational facilities increases the likelihood of perceiving the presence of these corresponding recreational facilities. Some of these associations depended on whether the existing recreational facilities were public, residential or commercial. With the exception of public cardio-training gyms, all types of public recreational facilities were associated with the corresponding facility perceptions, suggesting that more effort is needed to increase the awareness of public cardio-training gyms. Expectedly, the objectively-assessed number of residential table tennis courts, badminton courts and playgrounds for children had a greater effect (odd ratios) on facility perceptions than had their corresponding public recreational facilities. It is believed that the participants were more likely to correctly perceive the presence of residential facilities that were built near to the residences. The objectively-assessed number of public tennis courts and weight training gyms, however, had a greater effect on facility perceptions than had their corresponding commercial recreational facilities, indicating that participants were less aware of these types of commercial recreational facilities.

The non-significant results obtained through the use of GIS data involving the number of parks, pavilions, sports grounds, bike lanes, swimming pools and sports centres implied that simply assessing the number of these recreational facilities without categorizations of their usage location may not be sufficient to identify the determinants of MVPA. Parks, sports grounds and sports centres are multi-purpose recreational facilities that the features (e.g., costs of use) and availability of active facilities (e.g., number of basketball courts) vary from place to place. Although the number of parks and other multi-purpose recreational facilities have often been found to determine PA [39], the types of sport facilities that determined the associations remain unknown. Given that the features and active amenities available in the recreational facilities are also important attributes [20,40], it is suggested that future studies examine the features and availability of sport facilities within the parks, pavilions, sports grounds and sports centres in order to more precisely identify significant relationships with MVPA.

This study has several limitations. The cross-sectional nature of this study indicates that any cause and effect relationships could not be determined. It may be possible that active people choose to live in the activity-friendly neighbourhoods and have better perceptions of recreational facilities, rather than the environmental attributes influencing their MVPA. It is possible that the more active participants may have been more compliant in wearing the accelerometer than less active participants, limiting the generalizability of the finds. In addition, the direct observations conducted at one particular time and by one auditor may not precisely reflect the number of recreational facilities during the time when the MVPA of the residents was measured, in particular for the commercial recreational facilities which are vulnerable to closure from time to time. In addition, the 400 m radial buffer areas may not cover all the neighbourhood recreational facilities that are influence the residents, as people may access other recreational facilities located beyond the buffer areas when engaging in MVPA. The use of one-dimensional ActiGraph accelerometers on the hip may result in some types of MVPA being underestimated, including cycling, weight training and any activities that involve predominantly the upper body. Consequently, even though the perceived presence of cycling paths was associated with higher MVPA, this association may even have been higher if those participating in cycling as their main activity had their activity accurately measured by the accelerometer. This limitation for cycling is however unlikely to be very significant as the number of objectively measured bike paths in Hong Kong was very low (Table 1), and cycling on public roads is very uncommon in Hong Kong due to high vehicle density. Other small variations in the number of recreational facilities observed across different neighbourhoods may also have contributed to some of the non-significant results, especially for some types of residential and commercial recreational facilities that are not common in our selected neighbourhoods. The fact that only about 32% of the total participants provided valid accelerometry data may limit the generalizability of the findings, especially as further analyses (not reported here) showed that the education level and the employment status between those participants wearing and those not wearing the accelerometers were significantly different. Consequently, these findings cannot be generalized to the total 480 participants.

## 5. Conclusions

This study found that the perceived and objective measures of neighbourhood recreational facilities were associated with the residents’ MVPA and some associations depended on whether the recreational facilities were indoor or outdoor, public or residential. It is suggested that in future researchers categorize the recreational facilities into smaller divisions in order to identify significant determinants of MVPA. The objectively-assessed number of most types of public recreational facilities was associated with the corresponding facility perceptions, but the effect of public recreational facilities was generally lower than seen in the residential recreational facilities. While providing an activity-friendly physical environment is fundamental, public health policy makers should also improve the public awareness of recreational facilities inside the neighbourhoods in an attempt to promote MVPA. Further investigations are needed to examine the negative associations seen between MVPA and certain types of recreational facilities.
